# Dual embryonic origin of maxillary lateral incisors: clinical implications in patients with cleft lip and palate

**DOI:** 10.1590/2177-6709.20.5.118-125.sar

**Published:** 2015

**Authors:** Daniela Gamba Garib, Julia Petruccelli Rosar, Renata Sathler, Terumi Okada Ozawa

**Affiliations:** 1 Associate professor of Orthodontics, Universidade de São Paulo (USP), Hospital for Rehabilitation of Craniofacial Anomalies and School of Dentistry, Bauru, São Paulo, Brazil; 2 Student, Refreshment Course in Preventive and Interceptive Orthodontics, Universidade de São Paulo (USP), Hospital for Rehabilitation of Craniofacial Anomalies, Bauru, São Paulo, Brazil.; 3 Orthodontists, Universidade de São Paulo (USP), Hospital for Rehabilitation of Craniofacial Anomalies, Bauru, São Paulo, Brazil.

**Keywords:** Embryology, Cleft lip, Cleft palate, Incisor

## Abstract

**Introduction:**

Cleft lip and palate are craniofacial anomalies highly prevalent in the overall population. In oral clefts involving the alveolar ridge, variations of number, shape, size and position are observed in maxillary lateral incisors. The objective of this manuscript is to elucidate the embryonic origin of maxillary lateral incisors in order to understand the etiology of these variations.

**Contextualization:**

The hypothesis that orofacial clefts would split maxillary lateral incisor buds has been previously reported. However, recent studies showed that maxillary lateral incisors have dual embryonic origin, being partially formed by both the medial nasal process and the maxillary process. In other words, the mesial half of the lateral incisor seems to come from the medial nasal process while the distal half of the lateral incisor originates from the maxillary process. In cleft patients, these processes do not fuse, which results in different numerical and positional patterns for lateral incisors relating to the alveolar cleft. In addition to these considerations, this study proposes a nomenclature for maxillary lateral incisors in patients with cleft lip and palate, based on embryology and lateral incisors position in relation to the alveolar cleft.

**Conclusion:**

Embryological knowledge on the dual origin of maxillary lateral incisors and the use of a proper nomenclature for their numerical and positional variations renders appropriate communication among professionals and treatment planning easier, in addition to standardizing research analysis.

## Abstract (pt)

**Introdução:**

as fissuras de lábio e palato são malformações de alta prevalência na população. Nas fissuras que envolvem o rebordo alveolar, o incisivo lateral superior mostra variações de número, forma, tamanho e posição, o que o torna objeto de estudo, na tentativa de elucidar sua origem embrionária para compreender a etiologia dessas alterações.

**Contextualização:**

existia a hipótese de que a fissura orofacial seria capaz de segmentar o botão embrionário do incisivo lateral. No entanto, estudos recentes evidenciaram que o incisivo lateral superior possui dupla origem embrionária, sendo formado parcialmente pelo processo nasal medial e pelo processo maxilar. Em outras palavras, a metade mesial do incisivo lateral provém do processo nasal medial, enquanto a metade distal do incisivo lateral origina-se do processo maxilar. No paciente com fissura, não há fusão desses processos, o que resulta nos diferentes padrões numéricos e posicionais do incisivo lateral em relação à fissura. Além dessas considerações, propõe-se também uma nomenclatura para o incisivo lateral em pacientes com fissura labiopalatina, com embasamento na Embriologia, considerando-se sua posição em relação à fissura alveolar.

**Conclusão:**

o conhecimento embriológico da dupla origem do incisivo lateral superior e o emprego de uma nomenclatura adequada para as suas variações numéricas e posicionais facilita a comunicação entre profissionais, o planejamento dos casos e possibilita a realização de estudos clínicos comparativos.

## INTRODUCTION

Cleft lip and palate are craniofacial abnormalities characterized by the absence of continuity of maxillary structures^1^, exhibiting a multifactorial origin involving environmental and genetic factors.^2^ The prevalence of oral clefts is about 1 for every 1100 live births in the world, according to data from WHO.^3^ Due to the high prevalence of this craniofacial anomaly in the overall population, clinical implications of this condition demand attention during the rehabilitation process.

Cleft lip and cleft lip and palate determine a break in the alveolar ridge with an impact on the dentition and treatment prognosis.^4^ In unilateral complete cleft lip and palate, the maxilla is divided into two segments, one bigger and one smaller segment; in bilateral cleft lip and palate, the maxilla is segmented into three parts, two posterior segments and the pre-maxilla.^5^ Maxillary lateral incisors are critical teeth in individuals with clefts, considering the high prevalence of dental anomalies. Variations of number, shape, size and position are common.^6^^-^ 13 For this reason, several studies attempted to elucidate the embryonic origin of maxillary lateral incisor anomalies, so as to better understand the etiology of these phenotypic variations. 

This article aims to discuss, based on an embryological analysis, variations in number and position of maxillary lateral incisors in individuals with cleft lip and cleft palate, in addition to presenting a proposal of nomenclature for clinical and research usage and the clinical implications of cleft lip and palate patients' rehabilitation.

## LITERATURE REVIEW

### Embryology versus anatomy

The human face is developed during the first weeks of intrauterine life. The maxilla is embryologically constructed through the fusion of medial nasal processes which are centrally positioned on the face, in addition to bilateral maxillary processes laterally positioned.^14^ The fusion of these processes occurs bilaterally in the corresponding region of maxillary lateral incisors. When these facial embryonic processes fail to fuse, cleft lip or cleft lip and alveolus are established.^14^ The fusion of embryological processes is an event closely linked with the formation of the lateral incisor, both regarding time and location.^15^^,^ 16 ^,^ 17

In noncleft patients, anatomically, the maxilla consists not only of the pre-maxilla, region where the dental germs of the four incisors are located, but also of the maxillary posterior segments, where the dental germs of canines, pre-molars and molars are located. The pre-maxilla and the maxillary posterior segments are separated by a V-shaped incisive suture.^18^ Anatomically, the incisive suture is located between the lateral incisor and the canine, bilaterally.^6^

However, in patients with cleft lip and palate, a lateral incisor is commonly found in the posterior maxillary segment, distal to the alveolar cleft region and mesial to the maxillary canine, i.e., out of the pre-maxilla. How this occurrence is explained if lateral incisors are located in the pre-maxilla, mesial to the incisive suture, in patients without clefts? To make matters worse, some patients with orofacial clefts present a maxillary lateral incisor mesial to the alveolar cleft with or without the presence of a lateral incisor distal to the cleft. The question that arises is: Would the alveolar cleft split the maxillary lateral incisor tooth germ into two parts? What is the explanation for the location of a lateral incisor out of the pre-maxilla?

**Figure 1:**
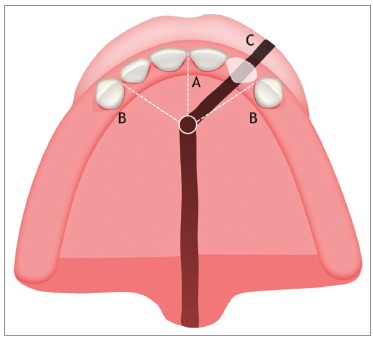
Illustrative image of the maxilla (occlusal view). A) Intermaxillary suture; B) Incisive suture; C) Location of the oral cleft.

In reality, there is no correspondence between the alveolar cleft location and the incisive suture (Fig 1).^6^^,^ 11 ^,^ 19 Animal embryology studies showed that incisive suture develop slightly distal to the region where the medial nasal process and the maxillary process fuse (Fig 1).^11^ And what is the relationship between this evidence and maxillary lateral incisor development? Despite lateral incisors being located anatomically in the pre-maxilla, its embryonic formation is dual. Maxillary lateral incisor mesial half originates from the medial nasal process (embryonic pre-maxilla) while its distal half originates from the maxillary process. Actually, after birth, the extreme mesial edge of the maxillary process will be located mesial to the incisive suture, composing the extreme distal region of the anatomical pre-maxilla.^6^^,^ 18

The hypothesis of dual embryonic origin of maxillary lateral incisors was corroborated by the study of Hovorakova et al.^18^ By means of tridimensional reconstructions, it was possible to observe two thickenings of dental epithelium being originated independently, one in the medial nasal process and another in the maxillary process, both separated by a narrow groove. The location of fusion of the dental epithelium was detectable in the form of a groove on the lateral incisor germ.^18^ The conclusion of this important study is that during prenatal development, the medial portion of the maxillary process gives in material for future anatomical pre-maxilla comprising central and lateral incisors.^18^ Thus, the mesial portion of the lateral incisor is probably originated in the medial nasal process (embryonic pre-maxilla) while this tooth distal portion must have been originated from the maxillary process.^18^^,^ 20

Corroborating the theory of dual embryonic origin of maxillary lateral incisors, a study analyzing monkeys' embryos with oral clefts found a union between the dental laminae of the medial nasal and maxillary processes forming an epithelial bridge, responsible for the formation of the embryonic lateral incisor tooth bud.^21^ A study evaluating the embryological formation of mice dentition found that a single tooth was formed through the fusion of two thickenings of dental laminae, which confirms the possibility of dual embryonic origin of maxillary lateral incisors.^22^

Considering this new evidence, the former hypothesis assuming that oral cleft could divide the tooth germ of maxillary lateral incisors^23^ is not confirmed. The hypothesis was based on the presence of two teeth between the central incisor and the canine on the cleft side, each one positioned at one side of the alveolar cleft. However, the idea that the embryonic button of lateral incisors was segmented into two parts improperly assumed that oral cleft occurred after the formation of the button, which is not true.^15^^,^ 23

**Figure 2:**
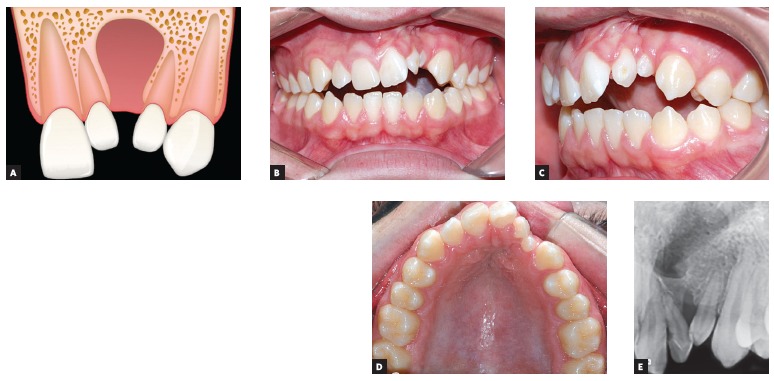
Maxillary lateral incisor mesial and distal to the alveolar cleft. A) Image of cleft illustrating the presence of two lateral incisors, one mesial and another distal to the alveolar cleft. B, C, D and E) Intraoral photographs and periapical radiograph of a patient presenting two maxillary lateral incisors at the cleft region.

**Figure 3:**
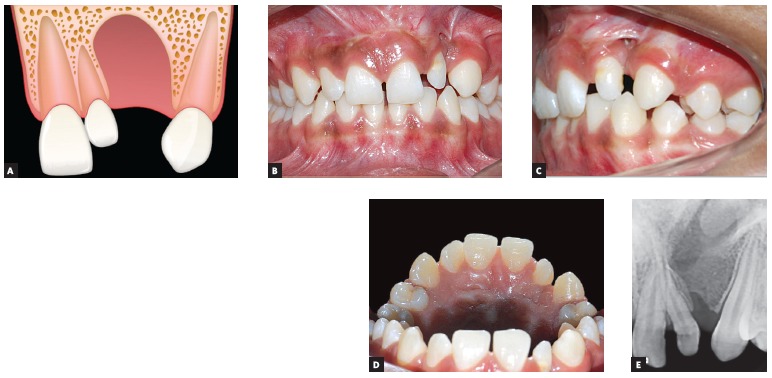
Maxillary lateral incisor mesial to the cleft: A) Image of the cleft illustrating the presence of only one maxillary lateral incisor mesial to the cleft. B, C, D e E) Intraoral photographs and periapical radiograph of a patient presenting only one lateral incisor mesial to the cleft region.

**Figure 4:**
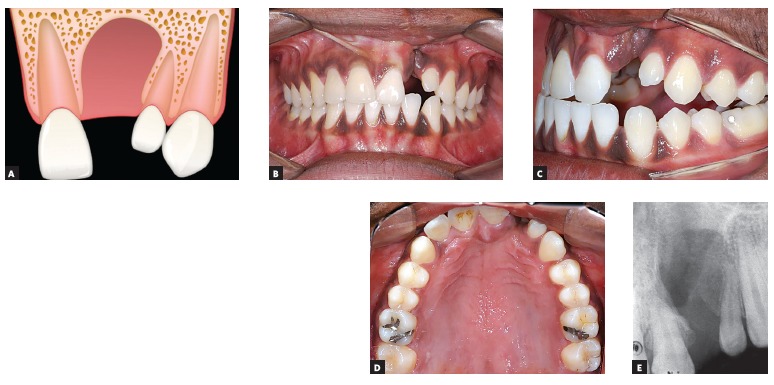
Lateral incisor distal to the cleft: A) Image of the cleft illustrating the presence of only one maxillary lateral incisor distal to the cleft. B, C, D e E) Intraoral photographs and periapical radiograph of a patient showing only a distal lateral incisor to the cleft region.

**Figure 5:**
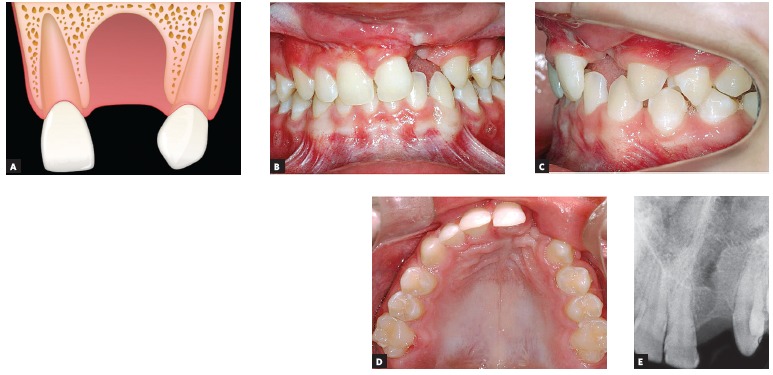
Agenesis of maxillary lateral incisor on the cleft side: A) Image of cleft illustrating absence of maxillary lateral incisor. B, C, D and E) Intraoral photographs and periapical radiograph of a patient presenting agenesis of maxillary lateral incisor on the cleft side.

With this in mind, the theory most accepted currently is the maxillary lateral incisor dual embryonic origin, with partial origin at the medial nasal process and partial origin at the maxillary process. This knowledge makes it possible to comprehend the high frequency of number anomalies shared by patients with clefts. In fact, there are four clinical numerical and positional situations regarding maxillary lateral incisors in the alveolar cleft region:

1. Presence of two maxillary lateral incisors, one mesial and the other distal to the alveolar cleft (Fig 2).
2. Presence of a single lateral incisor positioned mesial to the alveolar cleft (Fig 3).
3. Presence of a single lateral incisor located distal to the alveolar cleft (Fig 4).
4. Agenesis of maxillary lateral incisor adjacent to the cleft side (Fig 5): absence of lateral incisor both mesial and distal to the alveolar cleft.

The frequency of each one of these four occurrences varies according to the type of cleft and is compiled in Table 1. 

**Table 1:** Prevalence of numerical/positional situations for maxillary lateral incisors in patients with cleft lip/alveolus and complete cleft lip and palate.

	**Boehn^12^**	**Ranta^33^**	**Tsai^6^**	**Cassolato^28^**	**Pegelow^34^**	**Dentino^26^**	**Yatabe^20^**
	**(1963)**	**(1971)**	**(1998)**	**(2009)**	**(2012)**	**(2012)**	**(2013)**
Lateral incisor	n = 180	n = 83	n = 137	n = 116	n = 82	n = 141	n = 121
Mesial and distal	13.90%	31.30%	0.70%	17.20%	14.60%	13.50%	11.57%
Only mesial	13.90%	7.20%	1.50%	6.90%	7.40%	13.50%	9.09%
Only distal	30.60%	22.90%	46.00%	49.20%	39%	35.50%	38.84%
Agenesis	41.60%	38.60%	51.80%	26.70%	39%	37.50%	40.50%

### Nomenclature proposal

The current nomenclature for maxillary lateral incisors adjacent to the alveolar cleft is very confusing. There are several reports in the literature on the difficulties for research due to varied nomenclature and lack of a standardization.^24^^,^ 25

Some studies use the term "supernumerary" when there are two lateral incisors in the region of the cleft. Another denomination, dating back to 1973, is the term "pre-canine" to refer to lateral incisors distal to the alveolar cleft.^25^ This term was used to refute the idea that the tooth distal to the cleft was a lateral incisor, notwithstanding the shape similarity, only because the tooth was located outside the pre-maxilla. These nomenclatures were compatible with the knowledge available at that time.

**Figure 6:**
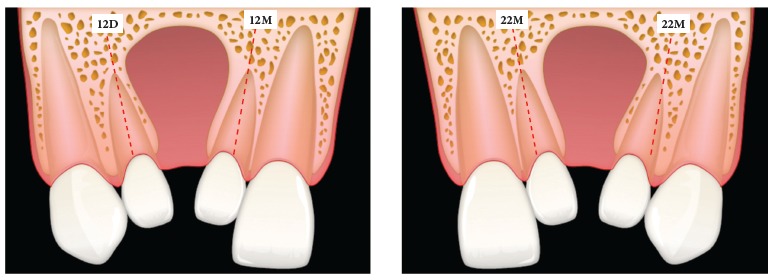
Nomenclature proposal for maxillary lateral incisors in patients with cleft lip and palate. Letters M and D refer to the mesial or distal location in relation to the alveolar cleft, respectively. Numbers 12 or 22 represent the lateral incisor positioned in the right or left hemimaxilla, respectively.

With the recent evidence of the dual origin of maxillary lateral incisors, the need of a new nomenclature was raised. Considering the four clinical variations of lateral incisors in patients with cleft lip and palate, as described in the previous topic, the use of the following nomenclature is proposed (Fig 6):

» Mesial lateral incisor (12M or 22M) and distal lateral incisor (12D or 22D) - these terms would be assigned to lateral incisors mesial and distal to the cleft, respectively. Therefore, the terms mesial and distal refer to the lateral incisor position in relation to the alveolar cleft. Figure 2 shows a patient with unilateral complete cleft lip and palate on the left side where both mesial and distal lateral incisors are present: the patient presents both 22M and 22D. In this case, there are two lateral incisors between maxillary central incisor and canine. Figure 3 illustrates a case of a patient with a left-sided cleft who presents only the mesial lateral incisor (22M). Differently, in Figure 4, only the distal lateral incisor (22D) is found on the cleft side (left side). Finally, in the case shown in Figure 5, neither the mesial nor the distal lateral incisor on the cleft side was present, which is considered tooth agenesis. 

In other words, this new nomenclature considers as maxillary lateral incisors any tooth located between maxillary central incisor and canine. The goal of standardizing the nomenclature for maxillary lateral incisors is to simplify the communication between professionals involved in rehabilitation of orofacial clefts as well as to make description in clinical or laboratory research in the field of molecular genetics easier.

## DISCUSSION

Agenesis is the most prevalent dental anomaly shared by cleft patients.^8^ Maxillary lateral incisors are the most affected teeth followed by maxillary and mandibular second premolars.^8^ Tooth agenesis is seen in both dental arches, not only on the cleft side, but also on the non-cleft side, probably due to an association between genetic factors and local factors related to the cleft.^26^

The most severe cases of oral clefts present higher prevalence of tooth agenesis.^12^^,^ 27 Previous studies showed that patients with unilateral complete cleft lip and palate had threefold frequency of maxillary lateral incisor agenesis on the cleft side than patients presenting unilateral cleft lip and alveolus.^24^^,^ 27 It is suggested that mesenchymal deficiency could be the possible explanation for non-fused facial embryonic processes, which results in cleft lip and palate.^6^ The etiology of cleft lip and complete cleft lip and palate seems to be similar, and the probable difference between them can be related to the degree of mesenchymal disability of embryonic processes. There is a hypothesis that the more hypoplastic embryonic tissues are, the greater the possibility of having a cleft palate established consecutively to cleft lip, and greater the probability of agenesis of permanent teeth.^24^

Different clinical patterns for maxillary lateral incisors in patients with oral clefts can be explained by the dual embryonic origin of these teeth. The presence of a single maxillary lateral incisor located mesial or distal to the cleft region can result in different degrees of hypoplasia of the embryonic processes involved in cleft formation.^18^ The presence of two maxillary lateral incisors on the cleft side, one mesial and one distal to the alveolar cleft, represents the independent development of two components of the lateral incisor embryonic button which were not united due to the cleft.^6^^,^ 18 The absence of maxillary lateral incisors can be explained by the severe mesenchymal disability in both medial nasal and maxillary processes, leading to absence of dental formation of both components of the lateral incisor.^6^^,^ 18

In Table 1, it is possible to observe that lateral incisor agenesis is the most frequent situation followed by the presence of only the distal lateral incisor (12D or 22D). The distal lateral incisor is more frequent than the mesial lateral incisor (Table 1) probably because hypoplasia is more frequent in the medial nasal process than in the maxillary process.^18^

What are the clinical implications of this new evidence and of the new proposed nomenclature? Orthodontic treatment is an important step in the rehabilitation process of the individual with orofacial cleft. In patients with agenesis of the lateral incisor on the cleft side (Fig 5), orthodontic correction may involve space closure by means of mesial movement of posterior teeth. In this case, the lateral incisor is replaced by the maxillary permanent canine after alveolar bone grafting,^28^ resulting in an adequate periodontal insertion of the canine moved to the grafted area.^29^ A second therapeutic option for lateral incisor agenesis is implant-supported prosthesis replacement in the posterior region of the dental arch. The decision between maintaining or closing the lateral incisor space depends on factors such as the position in which canines erupt, the extent of the cleft, tooth size discrepancy and the sagittal interarch relationship.^30^ Eventually, when the patient is not submitted to alveolar bone graft, it is possible to maintain the lateral incisor space for fixed or removable prosthesis rehabilitation. In non-grafted cases, besides prosthetic rehabilitation, there is the option of surgical movement of the posterior segment of the maxilla to close the space at the time of orthognathic surgery.^27^^,^ 30

Patients with at least one lateral incisor, mesial (12M or 22M) or distal (12D or 22D), as illustrated in Figures 3 and 4, respectively, can have comprehensive orthodontic treatment with lateral incisor maintenance, provided that adequate size and good periodontal insertion is observed. If necessary, prosthetic augmentation of the small lateral incisor crown can be performed after orthodontics.^30^^,^ 31

For patients with both maxillary lateral incisors (12M and 12D or 22M and 22D) on the cleft side, as shown in Figure 2, treatment plan is similar to non-cleft patients with supernumerary lateral incisor. One lateral incisor will be elected to dental extraction. In general, the incisor with shorter root or less bone insertion is chosen for extraction. 

The location of the maxillary lateral incisor in relation to the alveolar cleft directly influences the position of adjacent teeth. When the lateral incisor is mesial to the cleft, there is less space available at the pre-maxilla and, as a result, central incisors have a greater probability of erupting with rotations^32^. When the distal lateral incisor erupts at the palate, tooth extraction should be performed before secondary alveolar graft procedure in order to ensure an adequate palatal flap capable of covering the grafted area.^30^

In summary, the numerical variation of cases of maxillary lateral incisors in patients with oral cleft have several treatment plans that present good esthetic and functional results. When only one half of a lateral incisor is present adjacent to cleft, its preservation is important to maintain the alveolar bone in the cleft region, thereby improving the bone-graft prognosis and providing a good esthetic result.^30^

The dual origin of maxillary lateral incisors stimulates new hypotheses that should be evaluated in the near future. Within this perspective, the presence of two maxillary lateral incisors in patients without clefts could represent a mild expression of cleft lip and palate. In patients with isolated cleft lip, the frequent presence of two maxillary lateral incisors would represent an incomplete manifestation of the alveolar cleft.

## CONCLUSION

The dual origin of maxillary lateral incisors may justify the vulnerability for numerical and positional clinical variations. This study proposes a nomenclature that specifies the position of the lateral incisor in relation to the alveolar cleft. Two lateral incisor adjacent to the alveolar cleft can be observed, one mesial lateral incisor (12M or 22M) and one distal lateral incisor (12D or 22D). The proposed nomenclature contributes to the effectiveness of communication between the team during the rehabilitation of cleft lip and cleft palate besides standardizing research analysis.
